# Molecular characterization of colorectal cancer patients and concomitant patient-derived tumor cell establishment

**DOI:** 10.18632/oncotarget.7526

**Published:** 2016-02-20

**Authors:** Haa-Na Song, Chung Lee, Seung Tae Kim, Sun Young Kim, Nayoung K.D. Kim, Jiryeon Jang, Mihyun Kang, Hyojin Jang, Soomin Ahn, Seok Hyeong Kim, Yoona Park, Yong Beom Cho, Jeong Wook Heo, Woo Yong Lee, Joon Oh Park, Ho Yeong Lim, Won Ki Kang, Young Suk Park, Woong-Yang Park, Jeeyun Lee, Hee Cheol Kim

**Affiliations:** ^1^ Department of Medicine, Division of Hematology-Oncology, Samsung Medical Center, Sungkyunkwan University School of Medicine, Seoul, Korea; ^2^ Samsung Genome Institute, Samsung Medical Center, Seoul, Korea; ^3^ Department of Pathology and Translational Genomics, Samsung Medical Center, Sungkyunkwan University School of Medicine, Seoul, Korea; ^4^ Department of Surgery, Samsung Medical Center, Sungkyunkwan University School of Medicine, Seoul, Korea; ^5^ Department of Molecular Cell Biology, Sungkyunkwan University School of Medicine, Seoul, Korea; ^6^ Department of Internal Medicine, Gyeongsang National University School of Medicine, Jinju, Korea; ^7^ Department of Health Sciences and Technology, SAIHST, Sungkyunkwan University, Seoul, Korea

**Keywords:** colorectal cancer, patient-derived cell, somatic mutation

## Abstract

**Background:**

We aimed to establish a prospectively enrolled colorectal cancer (CRC) cohort for targeted sequencing of primary tumors from CRC patients. In parallel, we established collateral PDC models from the matched primary tumor tissues, which may be later used as preclinical models for genome-directed targeted therapy experiments.

**Results:**

In all, we identified 27 SNVs in the 6 genes such as PIK3CA (*N* = 16), BRAF (*N* = 6), NRAS (*N* = 2), and CTNNB1 (*N* = 1), PTEN (*N* = 1), and ERBB2 (*N* = 1). RET-NCOA4 translocation was observed in one out of 105 patients (0.9%). PDC models were successfully established from 62 (55.4%) of the 112 samples. To confirm the genomic features of various tumor cells, we compared variant allele frequency results of the primary tumor and progeny PDCs. The Pearson correlation coefficient between the variants from primary tumor cells and PDCs was 0.881.

**Methods:**

Between April 2014 and June 2015, 112 patients with CRC who underwent resection of the primary tumor were enrolled in the SMC Oncology Biomarker study. The PDC culture protocol was performed for all eligible patients. All of the primary tumors from the 112 patients who provided written informed consent were genomically sequenced with targeted sequencing. In parallel, PDC establishment was attempted for all sequenced tumors.

**Conclusions:**

We have prospectively sequenced a CRC cohort of 105 patients and successfully established 62 PDC in parallel. Each genomically characterized PDCs can be used as a preclinical model especially in rare genomic alteration event.

## INTRODUCTION

Colorectal cancer (CRC) is the third most common cancer in the world, with over 1 million cases diagnosed per year, and it is the fourth most common leading cause of cancer-related death, accounting for ∼8% of all deaths from cancer [1–3]. Given the significant incidence and mortality of CRC, tremendous efforts and resources have been dedicated to improve survival in patients with CRC, especially those with recurrent or metastatic disease. However, almost half of curative resected CRC cases ultimately relapse, and most of these cases remain refractory to salvage therapy, including systemic chemotherapy, palliative surgical resection, or radiation therapy [4]. Therefore, more effective therapies are needed to overcome the poor treatment outcome of CRC. Although several targeted agents have been discovered and are widely used in clinical practice, some patients do not respond to these targeted agents, emphasizing the need to develop more effective target therapies; this requires further investigation into the molecular characterization of CRC, including genomic analyses [5, 6].

Numerous molecular investigations have been carried out with the aim of developing effective targeted therapies. Such studies require suitable preclinical models that preserve the genomic integrity and accurately represent the biological characteristics of individual primary tumors [7, 8]. Examples of such preclinical models include cancer cell lines [9] or patient-derived xenograft (PDX) models [10–13]. However, these models also show some consistent disadvantages for use in clinical practice. Although cell line models are less costly than PDX models, they cannot accurately reflect the heterogeneity of primary tumors [14]. Such heterogeneity of the tumor and its microenvironment is captured in PDX models; however, these models are associated with difficulties in successful and rapid *in vitro* culture [15]. Alternatively, our previous study showed that patient-derived cells (PDCs) served as effective preclinical models, which may be less time consuming and more representative of the genetic diversity, heterogeneity, and drug sensitivity of tumors [16, 17].

In this study, we aimed to establish a prospectively enrolled CRC cohort for targeted sequencing of primary tumors. In parallel, we established collateral PDC models from the matched primary tumor tissues, which may be later used as preclinical models for genome-directed targeted therapy experiments.

## RESULTS

### CRC patient characteristics

From April 2014 to June 2015, we collected 112 CRC tissues for somatic mutation profiling and PDC cultures. The baseline demographic features of all patients are summarized in Table 1. The median age of the enrolled patients was 63 years (range, 25–88 years), who were diagnosed with colon cancer (80/112, 71.4%) or rectal cancer (32/112, 28.6%). Approximately half of all patients were diagnosed with stage IV (48/112, 42.8%), and most of them received palliative systemic chemotherapy (42/48, 87.5%). The majority of patients (80/105, 76.2%) were moderately differentiated (G2) type based on the histologic grade.

**Table 1:** Baseline clinical features of 112 CRC patients

Characteristics	No. of patients	(%)
**Age, year, median (range)**	63 (25–88)	
**Sex** (***N* = 112)**		
Male	60	53.6
Female	52	46.4
**Location (*N* = 112)**		
Colon	80	71.4
Rectum	32	28.6
**T stage (*N* = 112)**		
T1	2	1.8
T2	6	5.4
T3	71	63.4
T4	33	29.4
**N stage (*N* = 112)**		
N0	43	38.4
N1	29	25.9
N2	39	34.8
N3	1	0.9
**Stage (AJCC 7th edition) (*N* = 112)**		
I	4	3.6
II	28	25.0
III	32	28.6
IV	48	42.8
**Differentiation of cancer cell (*N* = 105)**		
Well differentiated (W/D)	12	11.4
Moderately differentiated (M/D)	80	76.2
Poorly differentiated (P/D)	13	12.4
**Presence of lymphatic invasion (*N* = 111)**	55	49.5
**Presence of vascular invasion (*N* = 111)**	28	25.2
**Presence of perineural invasion (*N* = 111)**	72	64.9
**Total examined nodes, Median (range)**	23 (1–75)	
**Number of positive nodes, Median (range)**	2 (0–28)	
**Status of K-ras mutation (*N* = 45)**		
Wild	24	53.3
12th codon mutation	19	42.2
13th codon mutation	2	4.5
**Status of EGFR expression (*N* = 45)**		
Negative	0	
1+ positivity	13	28.9
2+ positivity	25	55.6
3+ positivity	7	15.5
**Confirmation of BRAF (+) (*N* = 43)**	5	11.6
**Use of Cetuximab (*N* = 112)** at 1st post-operative chemotherapy	12	10.7
**Use of Avastin (*N* = 112)** at 1st post-operative chemotherapy	18	16.1
**Duration of follow up, month, median (range)**	11.02 (1.63–54.57)	

### Actionable genome profiling of the CRC patients cohort

**Figure 1:**
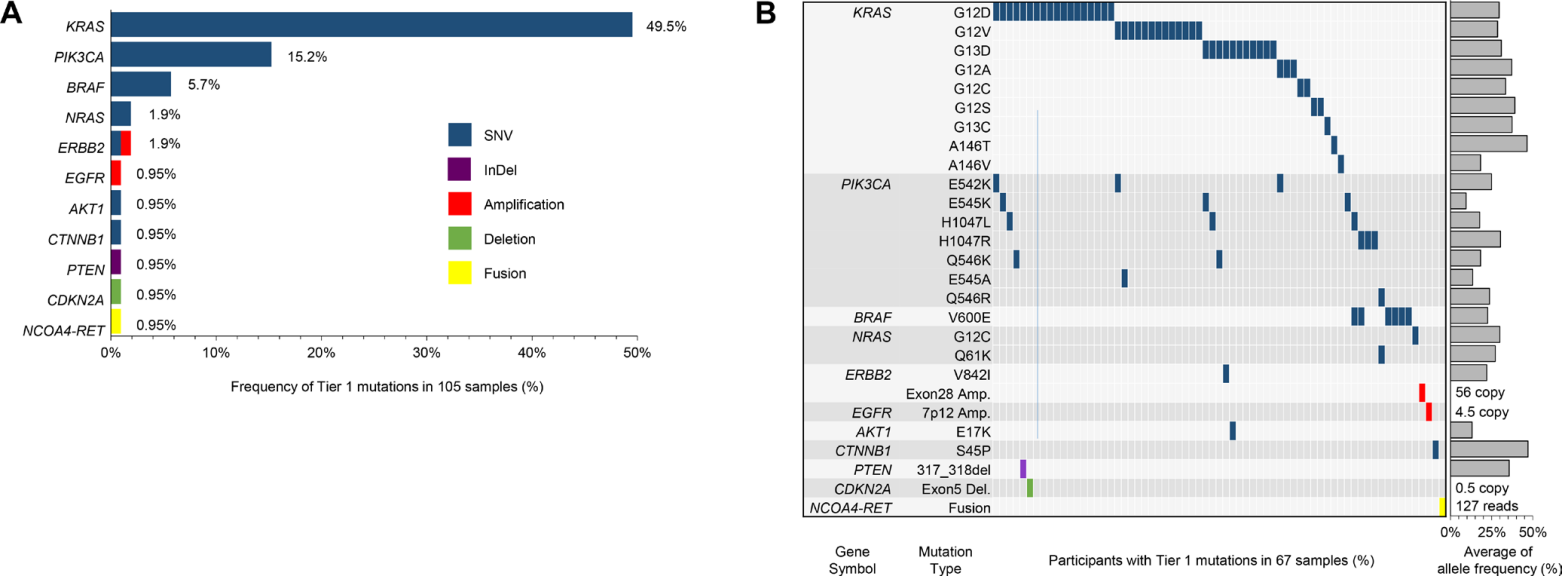
Somatic mutation profile of 105 CRC patients The targeted panel sequencing platform could identify “actionable” genome aberrations in 381 genes, including single nucleotide variations (SNVs), insertion and deletion (Indel), copy number variations (CNVs), and translocations. (**A**) represents Tier 1 mutations in 105 samples; (**B**) provides specific mutation sites for each gene.

Of the 112 patients enrolled, targeted sequencing was successfully completed for 105 patients due to the inadequate quality and low purity of tumor tissues. Mismatch repair (MMR) protein status in most of CRC patients (*N* = 102) were intact (microsatellite instable [MSI] low and sporadic subtype [L/S]), while three CRC patients showed high MSI subtype (MSI-H). The targeted panel sequencing platform could identify “actionable” genome aberrations in 381 genes, including single nucleotide variations (SNVs), insertion and deletion (Indel), copy number variations (CNVs), and translocations (Supplementary Table S1). About half of patients (49.5%) had recurrent somatic mutations in KRAS gene (Figure 1). We also identified 27 SNVs in the 6 genes such as PIK3CA (*N* = 16), BRAF (*N* = 6), NRAS (*N* = 2), and CTNNB1 (*N* = 1), PTEN (*N* = 1), and ERBB2 (*N* = 1). Most of driver mutations were present exclusively in CRC patients, especially BRAF and NRAS mutations were detected just in KRAS wild-type patients. On the other hand, PIK3CA mutations were more frequently found in KRAS mutation-positive patients (*N* = 10) than in KRAS wild type CRC patients (*N* = 6). PTEN and ERBB2 mutations were also observed in KRAS mutation-positive CRC patients.

With respect to CNVs, cyclin-dependent kinase 2A (CDKN2A) amplification was observed in one KRAS mutant-positive patient. Copy number amplification of ERBB2 and EGFR genes were observed in KRAS wild-type patients as well (Figure 1). RET-NCOA4 translocation was observed in one out of 105 patients (0.9%). The patient with the RET-NCOA4 translocation was 25 year-old male with no family history of CRC. Clinically he was at stage IV in pathological examinations and pT4aN2bM1 at diagnosis with poorly differentiated (G3) adenocarcinoma type. This tumor was found to be KRAS and BRAF wild type, and EGFR was not overexpressed. MMR protein was intact (MSI-S). He was treated with palliative chemotherapy with 8 cycles of a 5-fluorouracil (5FU)-based regimen; however, the treatment response was very poor and the patient showed a rapidly deteriorating clinical course.

### Primary tumor location and somatic mutations

**Figure 2:**
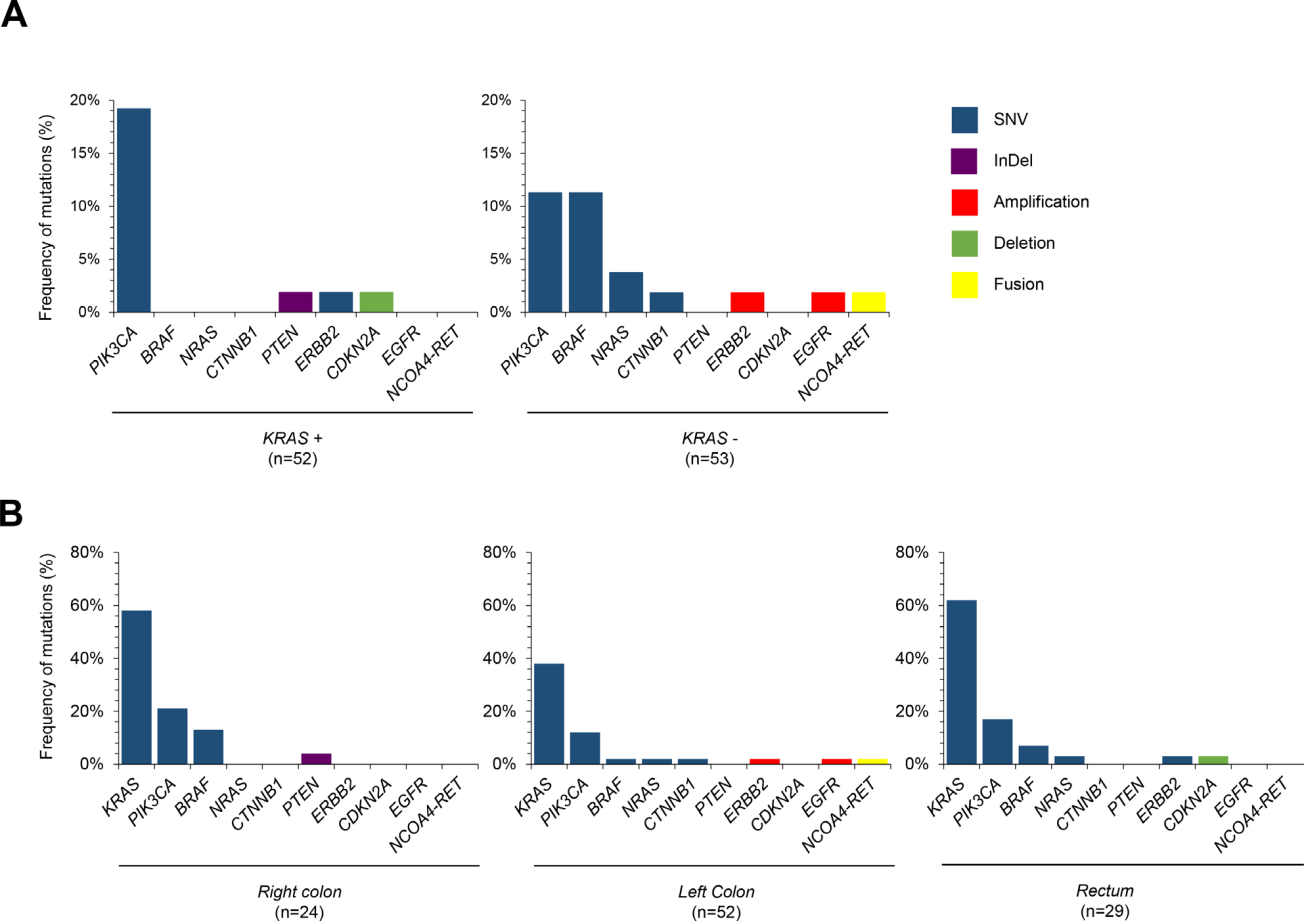
Primary tumor location and somatic mutations (**A**) Frequencies of genomic alterations according to KRAS mutation status in 105 CRC patients (**B**) Distributions of genomic alterations according to anatomic sites of primary tumor, right versus left versus rectal cancer.

We analyzed the genomic landscape of tumors according to the primary tumor location (right colon; left colon; rectum) (Figure 2). Anatomically tumors were taken from 24 (22.9%) cases of right colon, 52 (49.5%) cases of left colon, and 29 (27.6%) cases of rectum. There was no significant difference in the KRAS mutation frequency according to the primary anatomic site. A NRAS mutation (*N* = 1) was observed in a case of left colon cancer. ERBB2 mutation was observed in one rectal cancer patient, but not in cases of other anatomic sites of origin. Interestingly, the frequency of CNVs in the right colon was lower than that of patients in any other locations (Figure 2B).

### Molecular characteristics of PDC established from surgical specimens

**Figure 3:**
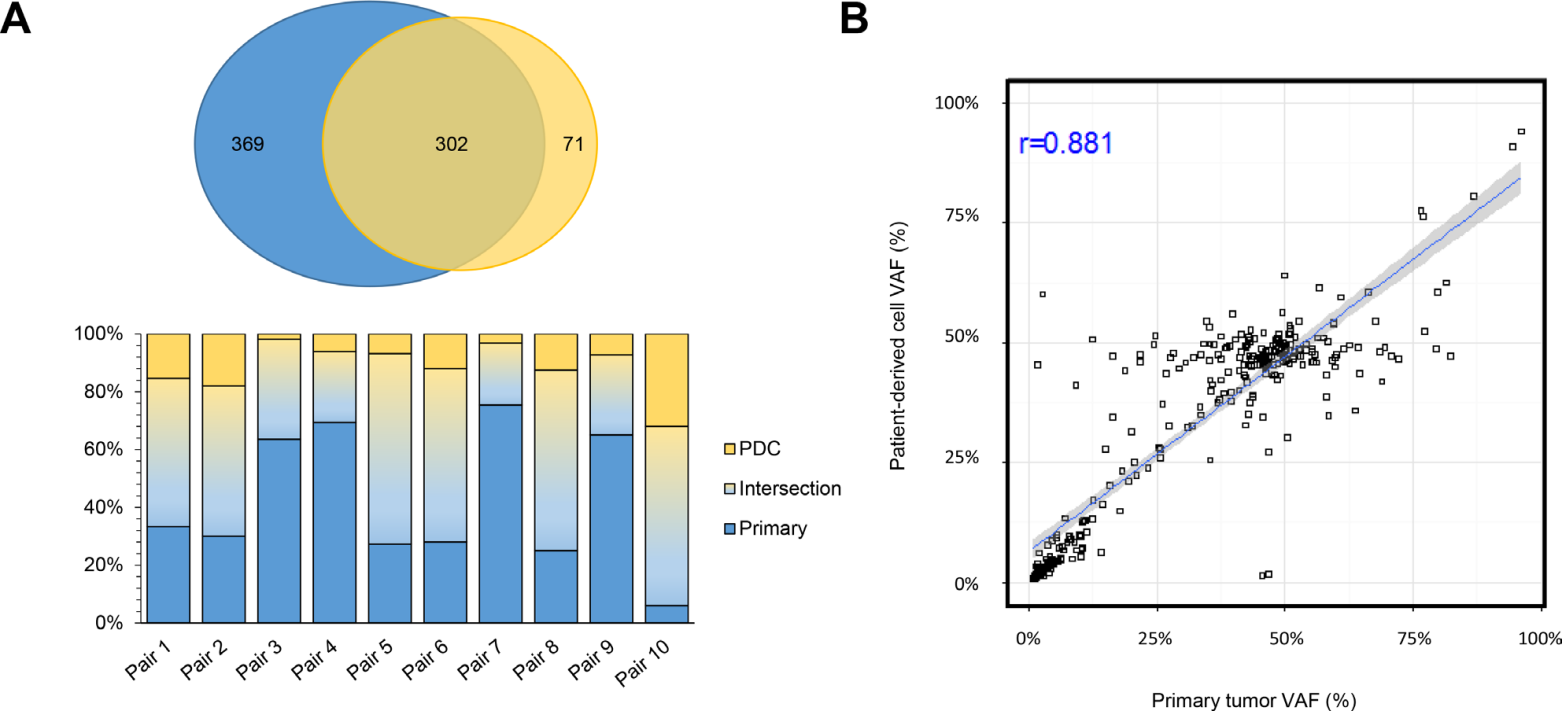
Comparison of genomic alterations between primary tumor and PDCs derived from primary tumor (**A**) Venn diagram showing the variants detected in the primary tumor and patient-derived cells (PDCs). Among 742 genomic alterations from 20 samples (10 primary and 10 PDCs), 302 were commonly detected from both types. (**B**) Correlations of variant allele frequencies (VAFs) between primary tumor PDCs. The plot shows VAFs of commonly identified SNVs and InDels from 10 samples. The Pearson correlation coefficient between the variants from primary tumor cells and PDCs was 0.881.

PDC models were successfully established from 62 (55.4%) of the 112 samples that were attempted. We defined the successful PDC according to the previous report [17]. First, we evaluated whether genomic alterations of the primary tumors were preserved in the cultured PDCs in 10 pairs of primary tumors and matched PDCs. Targeted sequencing of the 10 primary tumor–PDC paired samples revealed that genomic alterations were highly correlated with primary tumors (Figure 3).

**Figure 4:**
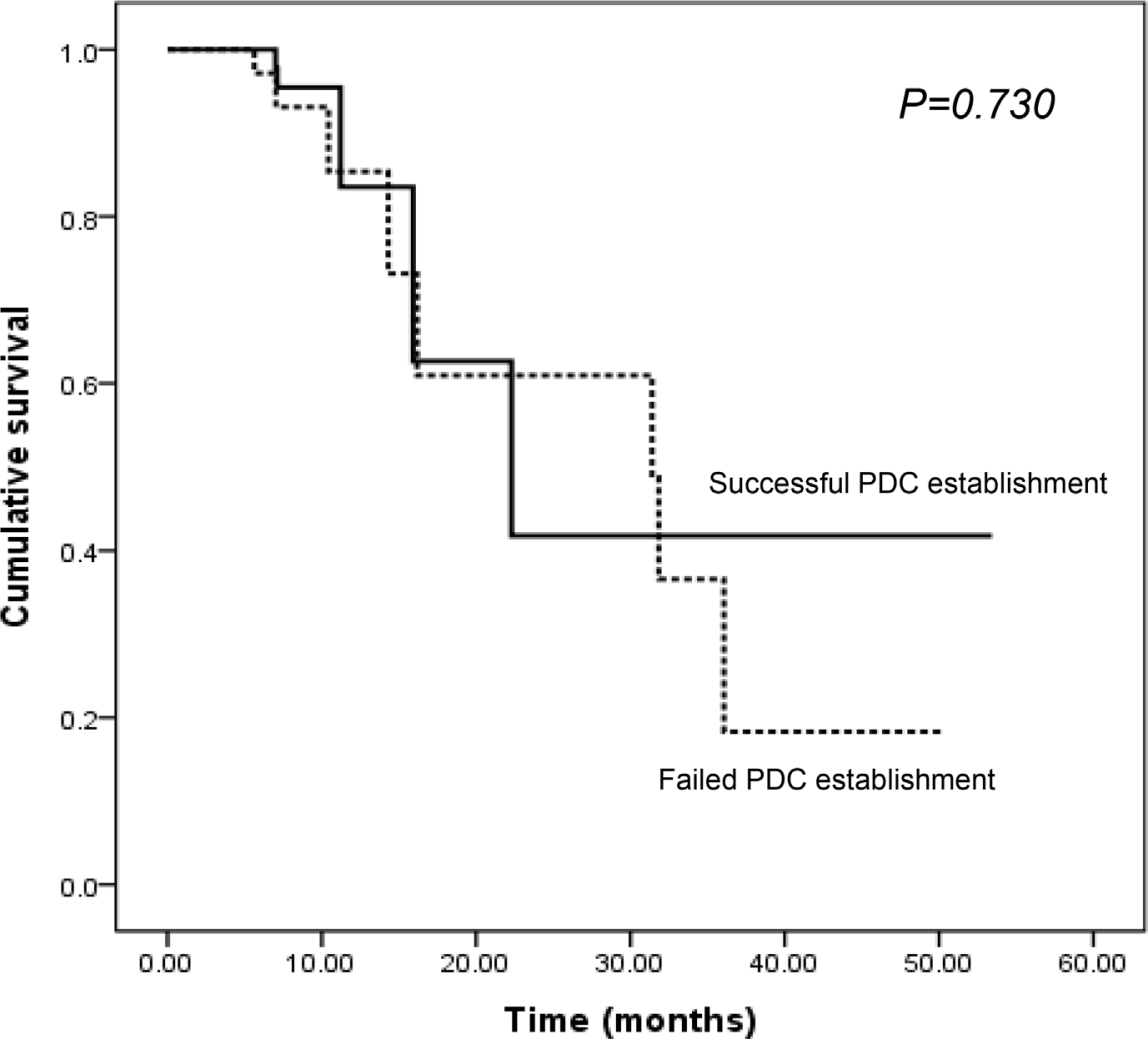
The impact of PDC establishment on PFS in CRC patients

Univariate comparisons revealed that the success of PDC establishment was significantly influenced by stage at diagnosis (stage I/II vs. III/IV: HR, 4.259, 95% confidence interval [CI], 1.743–10.410, *P* = 0.001) and the presence of vascular invasion (absence vs. presence: HR, 2.961, 95% CI, 1.172–7.479, *P* = 0.028) (Table 2). The location of the primary tumor, age, sex, histology of cancer cells, presence of lymphatic/perineural invasion, KRAS and BRAF mutation, or EGFR and ERBB2 expression did not significantly influence the success of PDC establishment. We analyzed the possible correlation between PFS and successful PDC establishment based on the hypothesis that more aggressive tumors with short PFS will render more likelihood to be grown *ex vivo*. However, progression-free survival (PFS) did not have a significant effect on the success of PDC establishment (median PFS 22.3 months vs. 31.4 months *P* = 0.730; Figure 4), although the follow-up duration was relatively short to draw any definitive conclusion (median FU duration: 11.02 months, range, 1.63–54.57 months).

**Table 2:** Univariate analysis for success of PDC establishment from surgical specimens

Characteristics	Hazard Ratio (95% CI)	*P* value
**Location of primary tumor (colon *vs* rectum)**	0.605 (0.265–1.382)	0.296
**Age (< 65 *vs* ≥ 65)**	0.533 (0.249–1.141)	0.125
**Sex (male vs female)**	1.458 (0.690–3.079)	0.348
**T stage (T1–3 *vs* T4)**	1.112 (0.492–2.513)	0.798
**N stage (N0, 1 *vs* N2, 3)**	1.863 (0.846–4.104)	0.123
**Stage (I, II *vs* III, IV)**	4.259 (1.743–10.410)	**0.001**
**Differentiation of cancer cell (W/D, M/D vs P/D)**	0.786 (0.245–2.518)	0.772
**Lymphatic invasion (absence *vs* presence)**	1.387 (0.657–2.930)	0.449
**Vascular invasion (absence *vs* presence)**	2.961 (1.172–7.479)	**0.028**
**Perineural invasion (absence *vs* presence)**	2.013 (0.912–4.441)	0.111
**KRAS (wt *vs* mt)**	1.00 (0.289–3.464)	1.000
**KRAS mt (12th *vs* 13th)**	0.632 (0.448–0.890)	0.533
**EGFR expression by IHC (1+, 2+ *vs* 3+)** **BRAF V600E (not detected *vs* detected)** **HER2 expression by IHC (negative, 1+ *vs* 2+, 3+)**	1.154 (0.195–6.820) 0.238 (0.035–1.639) 0.409 (0.050–3.367)	1.000 0.153 0.575

## DISCUSSION

A total 112 patients who were diagnosed with and underwent resection of CRC were enrolled in this study, and samples from 105 of these CRC patients were successfully sequenced with targeted sequencing. Of our patient cohort, 52 had *KRAS* mutation and 53 were *KRAS* wild type. Aside from KRAS mutations, the most commonly detected SNVs were in PIK3CA, BRAF, and NRAS, as described previously [18, 19]. Structural variations were relatively infrequently to find three cases of CDKN2A, ERBB2 and EGFR amplification and single one RET-NCOA fusion in a young patient with sporadic, left colon cancer (MSI-S). PDCs was successfully established from 55.4% (*N* = 62) of tumor specimens, and the genomic concordance rate between primary tumors and PDCs was very high. In this study, the incidence of KRAS (49%) and BRAF (5.7%) mutations was relatively higher than previous reports (43% and 3%, respectively) [20–23]. In addition, the frequency of PIK3CA mutation (15%) was comparable with that reported previously (15%); however, the NRAS mutation frequency (1.9%) was lower than previously reported data from 2,000 colorectal cancer patients in NSABP C-07 and C-08 trials (2.9%) [24]. Recently, the Alliance for Clinical Trials in Oncology has shown that BRAF and KRAS mutation frequencies are higher in White patients when compared with Asian CRC patients [22]. Moreover, most of the genomic sequencing in this study was performed on MSI-S CRC tumor specimens (97.1%) in our study. Because somatic mutation profiles in this study are reasonably similar to those of the Cancer Genome Atlas, CRC cases in this study can represent the clinical and biological characteristics of CRC.

Structural variation in CRC is an extremely rare event like ALK translocation which occurs in 0.8% of CRC cases [25] and NTRK1-TPM3 fusion in 0.2% of CRC samples [26]. These fusion events may provide an opportunity for the personalized therapy with targeted agents in this small subset of patients. For example, one report demonstrated that low-dose regorafenib, a novel RET inhibitor, potently suppressed RET fusion-positive CRC. Although an ALK immunohistochemistry assay has been validated as an effective screening tool, the correlation between RET fusion and RET immunohistochemistry was shown to be relatively low for some tumor types. Hence, targeted sequencing or a fusion-specific assay should be performed in order to identify this subset of CRC patients with the RET-NCOA fusion protein.

For developing individualized therapy, accurate prediction of the anti-tumor efficacy of novel agents is important, and the need for analysis of the genomic alterations of primary tumors has increased recently. Furthermore, preclinical models that closely resemble the genomic alterations observed in primary tumors have been investigated in numerous studies and are now widely used in several clinical fields. Ideal preclinical models will accurately reflect the genomic diversity and microenvironments of primary tumors, which would be valuable for testing novel target agents. Nevertheless, every preclinical model has inherent “tumor heterogeneity” issue since not all of the cells are 100% tumor cells or from the same clone of tumor (i.e. KRAS G12D tumor cell vs coexistent PIK3CA E542K tumor cell in the same tumor). Hence, we are currently conducting genomic sequencing of subsequent cell passages to parental tumor to investigate the extent of heterogeneity in PDC models. One of the major limitations of PDCs will be the lack of tumor microenvironment (i.e. stromal cells, tumor infiltrating lymphocytes) with the advent of immune-targeted therapy (i.e. pembrolizumab). However, PDCs are still useful to test the specific targets for each patient's tumor since more clinical trials are testing the targeted agents in combination with immune checkpoint inhibitors.

Few studies have evaluated the relationship between preclinical models and clinical outcomes in CRC [28, 29]. These studies have demonstrated that preclinical models such as the PDX models could be feasible for testing anti-tumor drug sensitivity. Moreover, one study showed that well-established PDCs can also be considered clinically useful models to demonstrate the sensitivity of novel targeted agents [30].

In summary, we have prospectively sequenced a CRC cohort of 105 patients and successfully established 62 PDC lines in parallel. We found commonly mutated SNVs as well as some rare CNVs and a rare fusion gene. We plan to massively screen these 62 PDCs established from the present CRC cohort with a panel of drugs using a high-throughput drug-screening platform with the aim of correlating drug sensitivity variations to the observed genomic aberrations.

## MATERIALS AND METHODS

### Ethics statement

The investigation has been conducted in accordance with the ethical standards of the Declaration of Helsinki and national and international guidelines, and has been approved by the Institutional Review Board at the Samsung Medical Center (SMC).

### Patient consent and study inclusion

Between April 2014 and June 2015, 112 patients with CRC who underwent resection of the primary mass, either in a palliative or curative setting, were enrolled in the SMC Oncology Biomarker study. Briefly, the inclusion criteria were as follows: age ≥ 18 years, pathologically confirmed colorectal cancer, and/or resection of the primary mass at the SMC. The PDC culture protocol was performed for all eligible patients. All of the primary tumors from the 112 patients who provided written informed consent were genomically sequenced. In parallel, PDC establishment was attempted for all sequenced tumors.

### MSI analysis

The MSI status was analyzed by PCR amplification using fluorescent dye-labeled primers for the Bethesda markers (BAT-26, BAT-25, D5S346, D2S123, and D17S250) specific to microsatellite loci, as recommended by the National Cancer Institute Workshop on MSI [31]. MSI was defined as a band shift in either of the two alleles, or as the appearance of a differently sized band in the tumor sample. Tumors were classified as MSI-H if instability was found in ≥ 50% of the loci screened, as MSI-L if at least one but ≤ 50% of the loci showed instability, and as microsatellite stable (MSS or MSI-S) if all loci were stable. Immunohistochemistry was used to detect the presence of the MMR proteins MLH1 and MSH2 in resected tumor specimens. For each antibody, a known MMR-positive normal colonic mucosa served as a positive control. Tumors known to lack MLH1 or MSH2 served as a negative control. All cases were scored as positive (defined as ≥ 10% of tumor cells stained) or negative (< 10% of tumor cells stained) for MLH1 or MSH2. The loss of MMR protein (MLH1 and/or MSH2) expression was defined as MMR-deficient (MMR-D), which is distinct from MMR intact (MMR-I). Tumors were considered to have MMR defects if they showed MMR-D expression and/or the MSI-H genotype. Tumors were considered to be MMR intact if they were MMR-I and/or showed the MSI-L or S genotype.

### DNA/RNA extraction

Genomic DNA was isolated using a QIAamp DNA Mini Kit (Qiagen, GmBH, Hilden, Germany), and total RNA was isolated with an RNeasy Mini Kit (Qiagen) according to the manufacturer instructions. The concentrations of genomic DNA and RNA were measured using a NanoDrop ND-100 spectrophotometer (Nano Drop Technologies, Wilmington, DE, USA). Genomic DNA and RNA were stored at −80°C.

### Targeted sequencing

Genomic DNA was extracted, and a SureSelect custom kit (Agilent Technologies, Santa Clara, CA, USA) was used for capturing information of 381 cancer-related genes. Illumina HiSeq 2500 was used for sequencing with 100-bp paired-end reads. The sequencing reads were aligned to the human genome reference sequence (hg19) using BWA-mem (v0.7.5), SAMTOOLS (v0.1.18), Picard (v1.93), and GATK (v3.1.1) for sorting SAM/BAM files, duplicate marking, and local realignment, respectively. Local realignment and base recalibration were carried out based on dbSNP137, Mills indels, HapMap, and Omni. SNVs and indels were identified using MuTect (v1.1.4) and Pindel (v0.2.4), respectively. ANNOVAR was used to annotate the detected variants. Only variants with an allele frequency greater than 1% were included in the results. The correlation coefficient was calculated based on variants that were detected in both cells.

### Primary cultures of surgical specimens

PDC cultures were established as previously described [25]. The tumors were homogenized, and extracted cells were cultured in RPMI medium supplemented with 10% FBS, 0.5 g/mL of hydrocortisone (Sigma Aldrich, St. Louis, MO, USA), 5 g/mL of insulin (PeproTech, Rocky Hill, NJ, USA), and 5 ng each of epidermal growth factor and fibroblast growth factor (PeproTech). The medium was changed every 3 days, and cells were maintained at 37°C in a humidified 5% CO_2_ incubator. PDCs were passaged using TrypLE Express (Gibco BRL) to detach cells when they reached 80–90% confluence.

### Cryopreservation of PDCs

Cells at 80–90% confluence were washed, detached using TrypLE Express, and incubated for 3 min at 37°C with 5% CO_2_. Following detachment, 4 mL of complete culture medium was added to block trypsin activity, and the cells were transferred to a 15- mL sterile centrifuge tube. After centrifugation, the cells were resuspended in 1 mL of freezing medium (CELLBANKER, Zenoaq, Japan), transferred into cryovials (Nalgene Nunc, Naperville, IL, USA), and frozen at −80°C overnight.

### Statistical methods

Standard descriptive and analytical methods were used to define the demographic and baseline clinical characteristics of the patient population. PFS was defined as the time from the date of surgery to the date of documented disease progression or death from any cause. Kaplan-Meier estimates were used in the analysis of the time-to-event variables, and the 95% CI for the median time-to-event was computed. Comparisons of survival by univariate analysis were estimated by the log-rank test. A *P*-value of less than 0.05 was considered statistically significant, and all *P*-values corresponded to two-sided significance tests. The statistical data were obtained using SPSS software version 18 (SPSS Inc., Chicago, IL, USA).
